# Subject-specific information enhances spatial accuracy of high-density diffuse optical tomography

**DOI:** 10.3389/fnrgo.2024.1283290

**Published:** 2024-02-19

**Authors:** Sruthi Srinivasan, Deepshikha Acharya, Emilia Butters, Liam Collins-Jones, Flavia Mancini, Gemma Bale

**Affiliations:** ^1^Department of Engineering, University of Cambridge, Cambridge, United Kingdom; ^2^Department of Psychiatry, University of Cambridge, Cambridge, United Kingdom; ^3^Department of Medical Physics and Biomedical Engineering, University College London, London, United Kingdom; ^4^Department of Clinical Neurosciences, University of Cambridge, Cambridge, United Kingdom; ^5^Department of Physics, University of Cambridge, Cambridge, United Kingdom

**Keywords:** optical neuroimaging, functional near-infrared spectroscopy, diffuse optical tomography, cortical parcellation, image reconstruction

## Abstract

Functional near-infrared spectroscopy (fNIRS) is a widely used imaging method for mapping brain activation based on cerebral hemodynamics. The accurate quantification of cortical activation using fNIRS data is highly dependent on the ability to correctly localize the positions of light sources and photodetectors on the scalp surface. Variations in head size and shape across participants greatly impact the precise locations of these optodes and consequently, the regions of the cortical surface being reached. Such variations can therefore influence the conclusions drawn in NIRS studies that attempt to explore specific cortical regions. In order to preserve the spatial identity of each NIRS channel, subject-specific differences in NIRS array registration must be considered. Using high-density diffuse optical tomography (HD-DOT), we have demonstrated the inter-subject variability of the same HD-DOT array applied to ten participants recorded in the resting state. We have also compared three-dimensional image reconstruction results obtained using subject-specific positioning information to those obtained using generic optode locations. To mitigate the error introduced by using generic information for all participants, photogrammetry was used to identify specific optode locations per-participant. The present work demonstrates the large variation between subjects in terms of which cortical parcels are sampled by equivalent channels in the HD-DOT array. In particular, motor cortex recordings suffered from the largest optode localization errors, with a median localization error of 27.4 mm between generic and subject-specific optodes, leading to large differences in parcel sensitivity. These results illustrate the importance of collecting subject-specific optode locations for all wearable NIRS experiments, in order to perform accurate group-level analysis using cortical parcellation.

## Introduction

Functional near-infrared spectroscopy (fNIRS) is a highly promising neuroimaging method, particularly for measuring brain activity during tasks performed in naturalistic environments, due to its portability and low sensitivity to movement compared to other neuroimaging modalities (Perpetuini et al., 2021). fNIRS non-invasively measures relative changes in oxygenated (HbO) and deoxygenated hemoglobin (HbR) using an array of light sources and detectors placed in contact with the scalp. Increased regional neuronal activity demands an increase in oxygenated blood flow to that brain region, which is faster than the rate of oxidative metabolism and thus leads to discernible increases in regional oxygenation (Phillips et al., 2016). As a result, the relative changes in blood oxygenation measured by fNIRS can be viewed as markers of brain activity (Yeung and Chu, 2022); this process is known as neurovascular coupling.

However, the lack of inherent structural information available from fNIRS data poses a challenge to performing valid comparisons of cortical activity across individuals with varying head shapes and sizes (Tsuzuki and Dan, 2013; Yücel et al., 2017; Zhai et al., 2020). More specifically, variability in the partial optical pathlength of fNIRS measurements across individuals has been previously attributed, in part, to differences in head structure (Nakamura et al., 2016; Cai et al., 2021). This in turn may lead to spurious differences in the magnitude of functional response observed between individuals.

High-density diffuse optical tomography (HD-DOT) is a neuroimaging method which builds on the concept of fNIRS by mapping three-dimensional changes in HbO and HbR concentrations that occur in superficial brain tissues. These three-dimensional images are produced by combining optical data with a light transport model derived from a structural prior of head anatomy (Hernandez-Martin and Gonzalez-Mora, 2020). In HD-DOT, a dense array of sources and detectors placed on the scalp is used to record light intensity from sampling overlapping volumes of tissue (i.e., overlapping channels) at varying source-detector separations. This can be used to yield information about the depth at which measured concentration changes occur (Wheelock et al., 2019) and permits the production of brain activity maps with spatial resolutions approaching those of functional magnetic resonance imaging (fMRI), as demonstrated in multiple studies (White and Culver, 2010; Eggebrecht et al., 2012, 2014; Ferradal et al., 2015).

As HD-DOT becomes more widely adopted, it is crucial to account for variability in cranial anatomy, as it can significantly impact the precise locations of optodes on the scalp surface (Tsuzuki and Dan, 2013). Overlooking such inadvertent shifts in optode locations may have important implications for research using HD-DOT. For example, the increased spatial resolutions achievable with HD-DOT offer the ability to perform finer-scale analysis of brain subregions using anatomical parcellation atlases (e.g., Fan et al., 2016; Gordon et al., 2016; Schaefer et al., 2018), however, this is assuming that the optode positions on the scalp surface can be correctly associated with the cortical surface underneath.

Previous work has demonstrated the importance of precise optode placement and accounting for anatomical variation in fNIRS. For example, multiple studies have investigated optimization methods for subject-specific optode placement guided by structural MRIs (Machado et al., 2018; Benitez-Andonegui et al., 2021). At the individual level, NIRS signal reproducibility was found to be improved when using an anatomical optode positioning approach (Novi et al., 2020), and in HD-DOT, individual variations in subject anatomy have been shown to impact image reconstruction quality (Zhan et al., 2012).

Studies that do not account for anatomical variation rely on the assumption that the same fNIRS array is sensitive to the same cortical regions across individuals. In this work, we use resting state data from multiple healthy adults to assess the degree of between-subject variability in cranial anatomy and HD-DOT array positioning, particularly on the underlying cortical regions to which we are sensitive. Photogrammetry was used to localize the optode placement on the scalp. This method involves using multiple overlapping images of an object, from different views, to determine the depth of points in a scene, allowing for the three-dimensional digital reconstruction of the imaged object. Prior work has demonstrated that photogrammetry-based approaches for optode localization yield lower localization errors in comparison to gold-standard electromagnetic digitizer methods (Mazzonetto et al., 2022). Numerous fNIRS and HD-DOT studies have also previously used photogrammetric optode registration methods for anatomical localization (Bluestone et al., 2001; Hu et al., 2020, 2021; Frijia et al., 2021; Vidal-Rosas et al., 2021; Uchitel et al., 2022, 2023).

Additionally, we investigate which brain regions suffer from the greatest error when optode positioning is assumed to be constant across all subjects. Although the error associated with localizing cortical activity in the absence of a subject-specific structural MRI (i.e., when using an anatomical brain atlas) has previously been found to be approximately double the error associated with doing so using subject-specific MRIs, atlas-guided methods for HD-DOT still demonstrate reasonable accuracy in localizing brain activity (Dehghani et al., 2009; Cooper et al., 2012). In this study, we thus use a template head atlas and instead focus on the minimization of other sources of error associated with the localization of cortical activity, namely the localization and registration of optodes to the head model. In doing so, we seek to demonstrate the ability to accurately localize cortical activity in HD-DOT recording settings, where the availability of subject-specific MRIs is not feasible.

## Materials and methods

### Participants

Prior to participant recruitment, a power analysis for a two-tailed test was conducted at 80% power (α = 0.05) to determine the sample size needed to reliably detect a difference between subject-specific and generic optode locations (Yücel et al., 2021). This was performed using data from an earlier pilot study (60s resting state recording, *n* = 22) where subject-specific photogrammetric data was available. To achieve a 10% minimum detectable difference between the two groups, a sample size of eight participants was deemed appropriate. Following this, ten healthy individuals were enrolled in this study (seven females and three males, aged 29.1 ± 7.2 years). All participants were informed about the experimental procedure and signed written informed consent forms prior to participating in the study. These studies were approved by the ethics committee within the Department of Engineering at the University of Cambridge.

### Data acquisition

During the experiment, an HD-DOT system (LUMO; Gowerlabs Ltd, London, UK) was used to record changes in detected light intensity across the region of interest. Here, we explore three regions of interest: the prefrontal, motor, and visual cortices. These regions were selected as they are the most widely interrogated in fNIRS studies (Huppert et al., 2009b). The HD-DOT system features a modular design, with hexagonal tiles that each contain three-dual wavelength sources (emitting at 735 and 850 nm) and four photodiode detectors (Zhao et al., 2021). The tile and optode layouts for each of the three recording regions can be found in Supplementary Figure 1. Two different cap designs with different tile layouts were used in this study, both fitted to a head circumference of 56–58 cm. The frontal cap featured 12 docks covering the prefrontal-frontal cortex while the motor/visual cap featured 30 total docks covering the parietal-temporal-occipital cortices. For the frontal and motor recordings, 12 tiles (36 sources and 48 detectors, 1,728 total channels) were used, while for the visual recording, six tiles were used (18 sources and 24 detectors, 432 total channels). Frontal recordings sampled channels at a rate of 12.5 Hz, while motor and visual recordings sampled channels at a rate of 5 Hz (due to constraints on data streaming). Data was recorded using a Dell Latitude laptop running LUMOx version 2.1.1. Experimental triggers were sent from the experimental presentation laptop to the recording laptop to mark the start and end of the measurement period.

### Experimental design

Each experimental session was carried out in a quiet room, with only the experimenters and participant present. The participant sat in front of a 13-inch MacBook Pro 2020 laptop, which presented the experimental instructions using PsychoPy (Peirce et al., 2019). In total, three 1-min resting state recordings, one for each region of interest (and thus tile layout), were taken per recording session (Geng et al., 2017). During these resting state recordings, participants were instructed to sit in a relaxed position while keeping their eyes closed. An auditory tone was played at the end of each 1-min period, after which the participant was allowed to open their eyes. The order of the three resting state recordings was varied across participants.

### Optode registration

**Figure 1:**
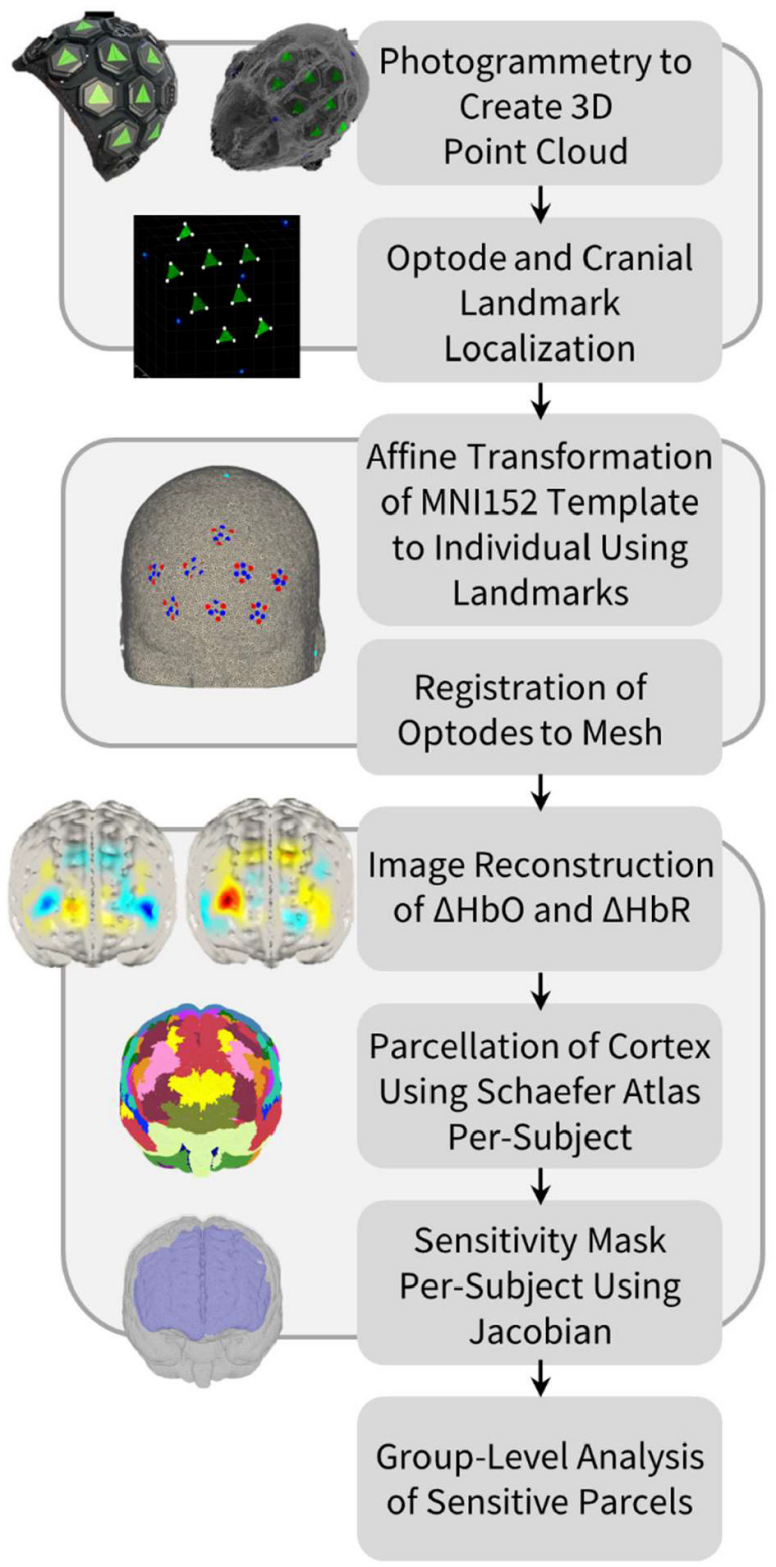
Processing pipeline for optode localization, head modeling and registration, image reconstruction of hemodynamic changes, and cortical parcellation in order to perform group-level anatomical analysis of the HD-DOT data.

A visual representation of the processing pipeline and its associated steps is shown in Figure 1. Photogrammetry was used to accurately identify the positions of optodes on the scalp surface. Since both the cap and tiles used are black, high-contrast green triangular stickers were attached to each tile, with each triangle corner overlying one of the three light sources on the tile (Vidal-Rosas et al., 2021). Additionally, high-contrast blue circular stickers were placed over five cranial landmark positions for each subject: the nasion (Nz), inion (Iz), left pre-auricular point (Al), right pre-auricular point (Ar), and vertex (Cz).

For each participant, two 360° panning videos of 10–15 s lengths were taken for each cap design using an iPhone XR (Apple Inc., Cupertino, USA), while the participant was seated with their eyes closed. One video was taken at approximately eye-level while the second was taken slightly above eye-level to ensure that all tiles and cranial landmarks were captured. Approximately 60 equally spaced frames were extracted from each video as still images and imported into Metashape photogrammetry processing software (Agisoft LLC, St. Petersburg, Russia). For each cap design, a three-dimensional model of the participant's head, with tile and cranial landmark markers visible, was produced and exported as a point cloud. A program written in MATLAB R2022b (MathWorks Inc., Natick, USA) allowed for the high-contrast stickers to be isolated according to their RGB color values. Based on these values, the locations of each source and cranial landmark were approximated, and the detector locations could then be identified using known tile geometries.

### Data pre-processing

The HD-DOT data were pre-processed using the Homer2 fNIRS analysis toolbox (Huppert et al., 2009a). Raw intensity data were first converted to changes in optical density. Channels were discarded if their coefficient of variation (standard deviation of intensities/mean of intensities) was above 8%, their source-detector separation was above 100 mm, or the mean signal intensity was > 1 × 10^11^ (Frijia et al., 2021; Vidal-Rosas et al., 2021; Zhao et al., 2021). Motion correction was not performed in this study, as the motion burden was determined to be low (between 10 and 15% of the total recording) for all sessions. Motion burden was assessed using the Homer2 function *hmrMotionArtifact*. The data were then visually inspected during the resting state period to ensure that there were no motion artifacts present. Optical densities were converted to HbO and HbR concentration changes, and a fifth-order Butterworth bandpass filter was used (cut-off: 0.01–0.2 Hz) on each channel (Pinti et al., 2019). The DOT-HUB Toolbox (https://github.com/DOT-HUB/DOT-HUB_toolbox) was used to perform local short channel regression (Uchitel et al., 2022). In this case, data from the nearby short-separation channels (<12 mm) were used to regress out systemic interference in each long channel, thus taking into account the localized variations in extracerebral signals (Wyser et al., 2020).

### Head modeling and image reconstruction

A four-layer mesh model of the head—mapping the spatial distribution of gray matter, white matter, cerebrospinal fluid, and extra-cerebral tissue—was used for all participants. This head model was produced using the MNI152 atlas (Mazziotta et al., 1995), which was derived from the MRI scans of 152 healthy individuals, averaged together after registration to the common MNI coordinate space (Mandal et al., 2012). The four-layer head mesh model was created using the Iso2Mesh mesh generation toolbox in MATLAB (Fang and Boas, 2009). This head model was then warped to the native space of each subject via an affine transformation between the five cranial landmarks (Nz, Iz, Ar, Al, Cz) of the head model and the photogrammetry-derived cranial landmarks of a given subject. Once the appropriate affine transformation was determined, the subject-specific optode array could be registered to the head model.

To quantify head size across participants, two distance metrics based on the cranial landmark positions were used: nasion-inion distance and distance between pre-auricular points. The shortest path between the two relevant points on the head model, constrained to passing through Cz, was calculated using the three-dimensional Fast Marching algorithm (Deschamps and Cohen, 2001). Once this path was known, the geodesic distance between the relevant points could be calculated as the arclength of the curve. The head size distribution was tested for normality using the Kolmogorov-Smirnov test.

The Toast++ software toolbox was used to perform image reconstruction (Schweiger and Arridge, 2014). A forward model of light propagation from source to detector for each channel was computed using the diffusion approximation to the radiative transfer equation. A Jacobian matrix, whose matrix elements represent measurement sensitivity to small changes in optical properties, was then calculated per-wavelength using the finite element method (Schweiger et al., 1993). The inverse of the Jacobian was calculated using the Moore-Penrose method, and a zeroth-order Tikhonov regularization was performed with a regularization hyperparameter of 0.01 (Wang et al., 2023). Following this, reconstructed images of changes in HbO and HbR concentrations were derived at each time point for each node of the gray matter mesh. The DOT-HUB Toolbox was used to formulate the forward model and perform the inversion.

### Cortical parcellation

The cortex of the head model was parcellated into distinct anatomical regions, based on the approach taken by Uchitel et al. (2022). This enabled direct cross-participant comparisons to be made regarding the cortical regions to which our HD-DOT array was sensitive. For the purpose of this study, the highest resolution Schaefer cortical parcellation atlas (1,000 parcels) was used, with each parcel matched to one of the 17 Yeo resting-state functional networks (Yeo et al., 2011; Schaefer et al., 2018). This parcellation atlas was already registered to the volumetric MNI space and could thus be directly converted from voxel positions to node locations. Each node in our standard gray matter surface mesh, previously derived from our atlas head model, could then be matched to its nearest node in the parcellation atlas. Using the Jacobian matrix, we defined the gray matter nodes to which the array was sensitive as those that have any channels with a sensitivity value above 5% of the maximum sensitivity value for that channel, across both wavelengths (Uchitel et al., 2022). Thus, for a parcel to be included, it needed to demonstrate sufficient sensitivity at both wavelengths. As a result, for any given participant, the specific parcels to which our HD-DOT array was sensitive do not vary across chromophores (HbO and HbR).

### Comparison of generic and subject-specific array sensitivity

Using the method described above, differences in array sensitivity were assessed using both generic optode locations and the subject-specific optode locations that were derived using photogrammetry. The generic optode locations and generic cranial landmarks positions were measured using a Polhemus PATRIOT™ digitizer system (Polhemus, USA) from a phantom adult head on which the cap was placed. This process took place during device manufacture and was applied to all participants during pre-processing. Given the rigid geometry of each tile, the exact within-tile distance between sources and detectors (either 10 or 20 mm) is known (Frijia et al., 2021).

All pre-processing and image reconstruction steps were kept the same between the two methods. To assess whether there were significant differences in optode localization errors between the three head regions and between hemispheres, a two-sided Wilcoxon signed rank test was used for each paired comparison. Scattered data interpolation between optode locations was performed using a Delaunay triangulation to generate a smooth map of optode localization errors (Amidror, 2002), with extrapolation to all points in the scalp surface mesh that were within 20 mm of an optode.

### Individual- and group-level parcellation analysis

Parcel sensitivity percentages were calculated for each subject, at the individual level, using both subject-specific and generic optode locations. For the purpose of our analysis, we report parcel sensitivity as the percentage of gray matter surface mesh nodes that the NIRS array was sensitive to for a given parcel. Parcels were then included in the group-level analysis if at least six out of eight participants (75%) demonstrated any sensitivity (>0%) to any nodes in the same parcel. Mean parcel sensitivity percentages across participants were calculated for these included parcels.

Afterward, group-level parcellation maps were converted to binary images classifying whether a parcel was included or not. The Jaccard index was determined for these greyscale group-level parcellation maps as a means of quantifying the similarity between parcellation maps calculated using generic optode locations and subject-specific optode locations. A Jaccard index of 1 indicates that the two parcellation maps are in total agreement, while a Jaccard index of 0 indicates that there is no overlap between the parcellation maps. Additionally, the HbO values for all parcels that we were sensitive to were determined as the average HbO value across all nodes within that parcel at a given time point. Paired *t*-tests were used to compare the difference between subject-specific and generic HbO parcel values at both the individual and group levels, and false discovery rate (FDR) correction was used to adjust for multiple comparisons.

## Results

Of the ten HD-DOT datasets collected as part of this study, errors in data acquisition occurred for two participants, leading to their datasets being excluded from the parcellation analyses due to very low signal-to-noise ratios (≪8% for all channels between 20 and 40 mm) across channels. Thus, for the parcellation analysis, our final sample size was comprised of eight healthy adults (five females and three males, aged 28.8 ± 7.9 years). However, cap placement data for all ten original participants were still utilized in all analyses that solely required optode location information. The distribution of head sizes across all ten original participants was found to be normally distributed (Nz-Iz distance: 37.3 ± 1.9 cm, Ar-Al distance: 37.8 ± 2.2 cm). Supplementary Table 1 provides additional detail on these metrics for each subject. Additionally, because the parcel sensitivity maps are independent of chromophore (HbO and HbR), we have presented our HD-DOT array sensitivity results in the context of HbO, with HbR results being consistent.

### Region-specific offset in optode location

**Figure 2:**
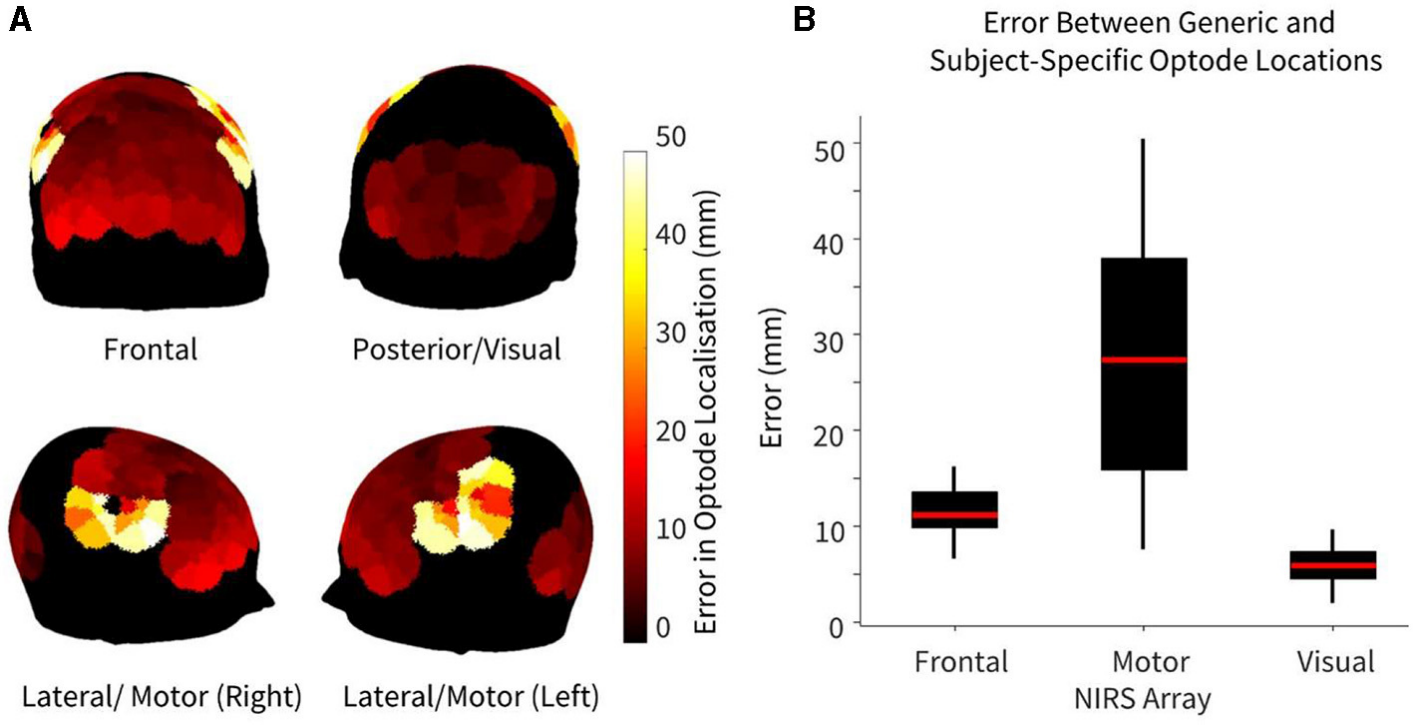
Error between generic and subject-specific optode locations, where error is defined as the Euclidean distance between optode (source/detector) locations using both methods, shown for *n* = 10 participants. **(A)** Heatmap of median difference in optode locations using both generic and subject-specific positions. **(B)** Error in optode localization for each NIRS array. For arrays that had >6 tiles (frontal and motor), only the six tiles with maximum error (three per hemisphere) were chosen to avoid biasing the boxplot.

The error in optode location, defined as the Euclidean distance between generic and subject-specific HD-DOT optode arrays, is summarized in Figure 2. Maximum errors between generic and subject-specific positions were found overlying the motor cortex, particularly on the lateral regions of the head (Figure 2A). Three tiles in both the left and right hemispheres overlying the motor cortex demonstrated the highest error, with a median error of 27.4 mm (IQR: 15.9–37.9 mm). For the frontal recordings, the largest errors were found overlying the lower frontal region, above the brow ridge, with a median error of 16.3 mm (IQR: 9.9–13.6 mm). The visual recordings demonstrated the lowest error between the generic and subject-specific optode locations (median error = 5.9 mm, IQR: 4.5–7.4 mm). Statistically significant differences in optode location errors were found across all three HD-DOT array layouts at the *p* < 0.05 significance level, indicating region-specific optode localization errors. No significant hemispheric differences in localization error were found in any of the three recordings.

### Individual- and group-level differences in HD-DOT array sensitivity

Large participant variations were found in the cortical sensitivity of the same HD-DOT tile layout. Thus, only parcels that ≥75% of participants were sensitive to were included in the parcellation analysis. Throughout, we refer to the abbreviated network names as defined by Yeo et al. (2011). However, it is important to note the proper functional networks that these correspond to. As such, the Control A and Control B networks refer to the frontoparietal control network and the Default A, B, and C networks refer to the default mode network.

Surprisingly, though the optode localization error for the visual recordings was the lowest of the three HD-DOT array layouts, the Jaccard index (range: 0–1), which was used to measure similarity between the generic and subject-specific parcel sensitivity maps at the group-level, was found to be lower (0.76) than that of the frontal recordings (0.82). The Jaccard index for the motor recordings was the lowest of the three layouts (0.73), signifying the least agreement between parcellation maps.

**Figure 3:**
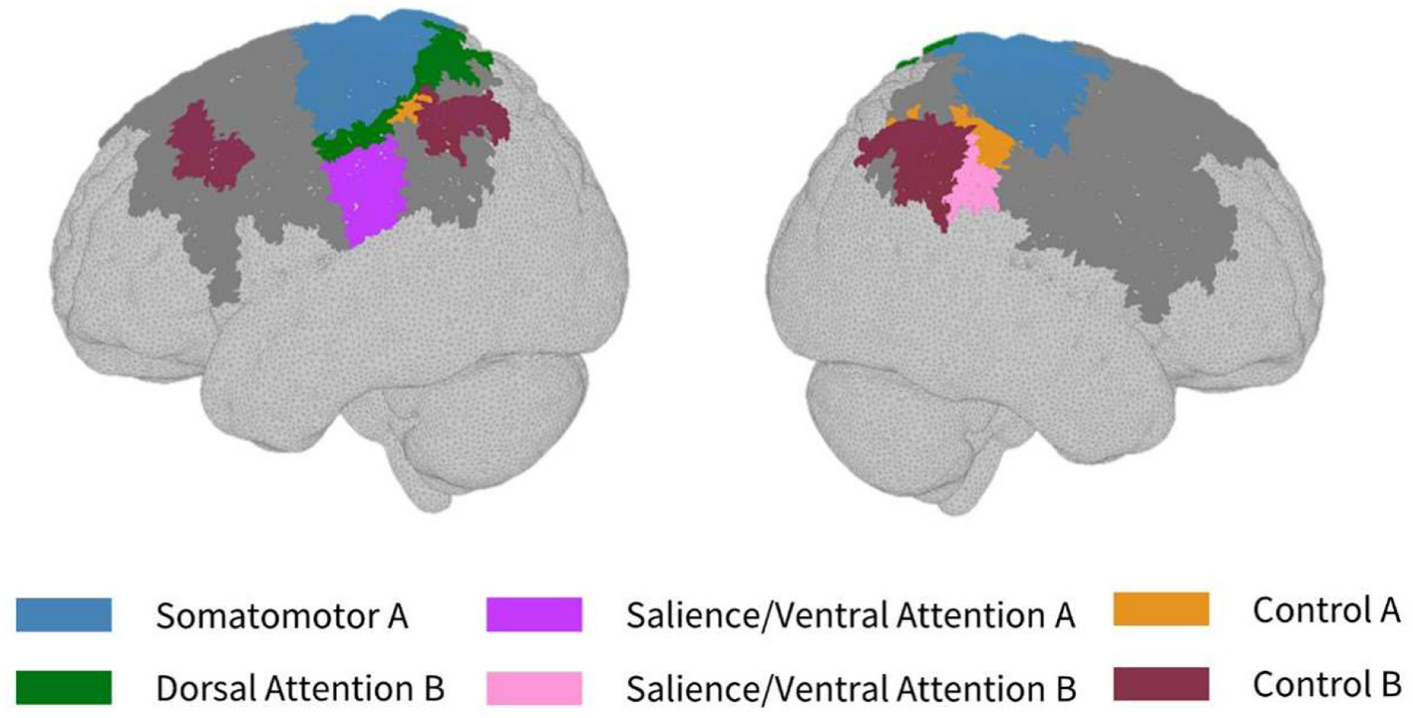
Maps of sensitivity differences between the subject-specific and generic optode locations for the motor cortex (lateral view), shown for *n* = 8 participants. Dark gray areas represent the maximum area we can be sensitive to across all participants using the motor HD-DOT array layout, derived from the union of parcels in the individual parcel sensitivity maps (both subject-specific and generic). Colored parcels are those that showed >10% difference in parcel sensitivity across at least half (4/8) of the participants. Parcel colors refer to the functional network they belong to.

**Figure 4:**
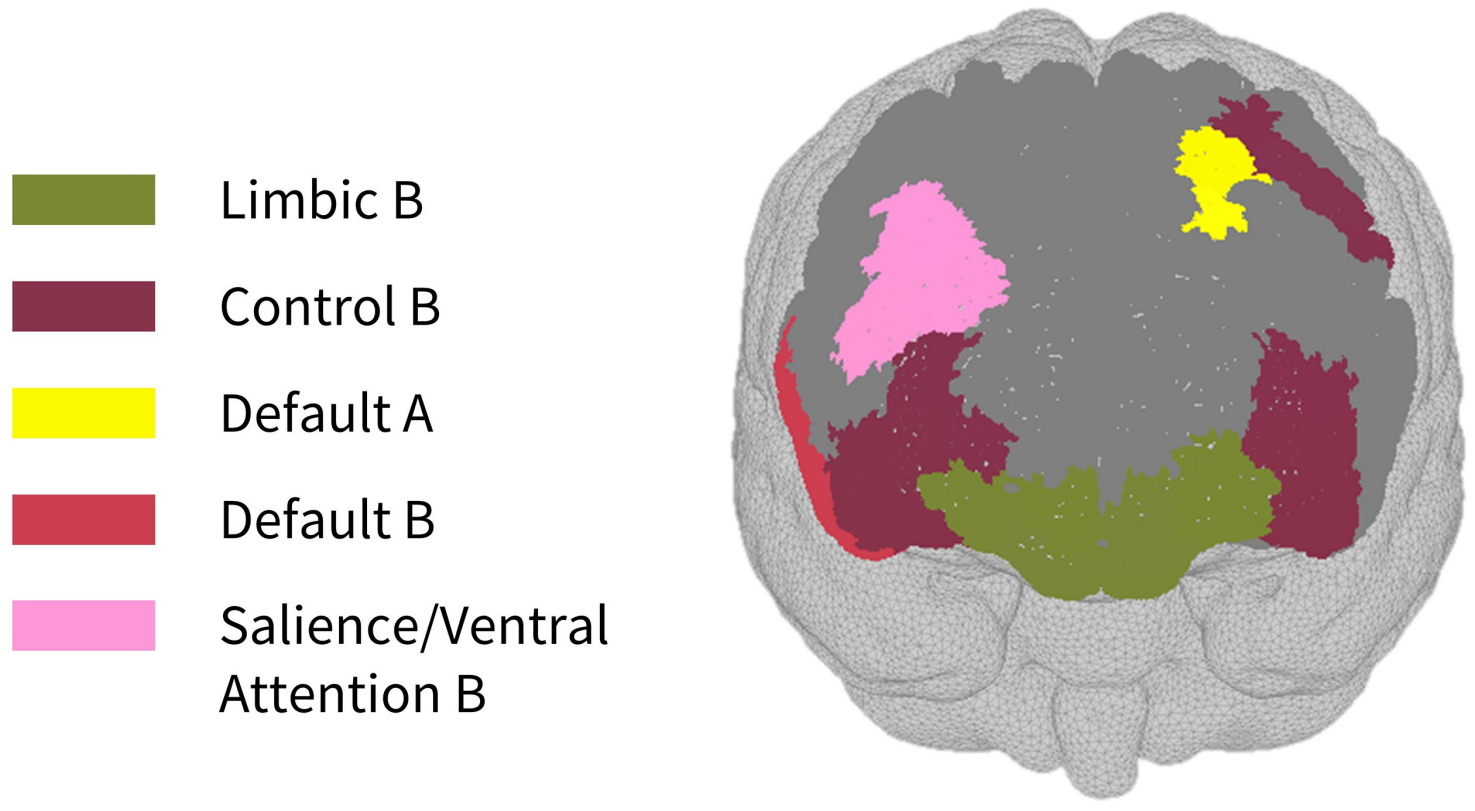
Map of sensitivity differences between the subject-specific and generic optode locations for the prefrontal cortex (frontal view), shown for *n* = 8 participants. Dark gray areas represent the maximum area we can be sensitive to across all participants using the frontal HD-DOT array layout, derived from the union of parcels in the individual parcel sensitivity maps (both subject-specific and generic). Colored parcels are those that showed >10% difference in parcel sensitivity across at least half (4/8) of the participants. Parcel colors refer to the functional network they belong to.

**Figure 5:**
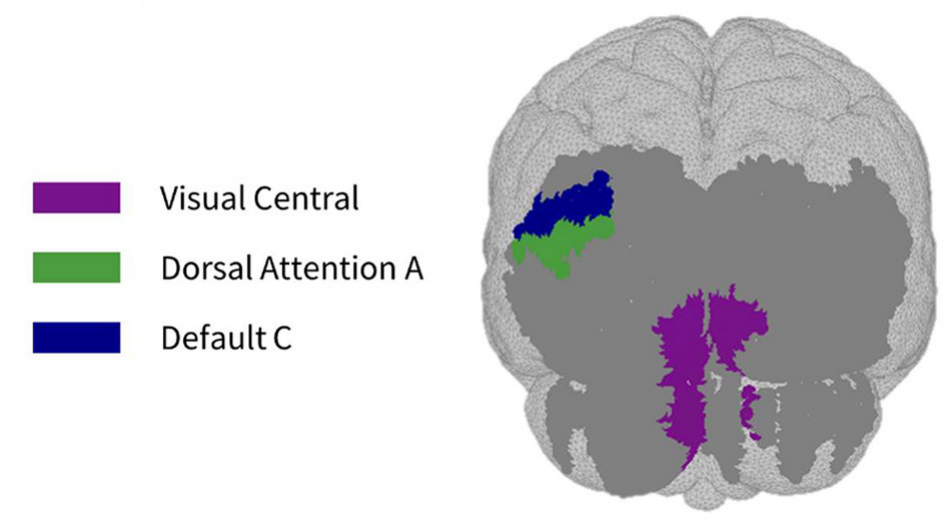
Maps of sensitivity differences between the subject-specific and generic optode locations for the visual cortex (posterior view), shown for *n* = 8 participants. Dark gray areas represent the maximum area we can be sensitive to across all participants using the visual HD-DOT array layout, derived from the union of parcels in the individual parcel sensitivity maps (both subject-specific and generic). Colored parcels are those that showed >10% difference in parcel sensitivity across at least half (4/8) of the participants. Parcel colors refer to the functional network they belong to.

As seen in Figure 3, most parcels demonstrating >10% difference in sensitivity between the subject-specific and the generic arrays for the motor recordings occurred in the posterior of the total cortical surface we were sensitive to. Overall, the motor HD-DOT array was reliably sensitive to parcels in six bilateral networks. Additionally, the mean absolute percentage difference in sensitivity was greatest for the motor recordings (19.42 ± 19.30%), which was in line with the optode localization errors in this area. For the frontal and visual recordings, the mean absolute percentage difference in sensitivity was lower (14.70 ± 12.00% and 14.23 ± 15.14%, respectively). For the frontal array, parcels in five bilateral networks were identified, with the lower prefrontal cortex showing the largest differences in sensitivity between the two optode localization methods, as seen in Figure 4. This was again in line with our findings for the optode localization errors in this area. The visual array demonstrated reliable sensitivity to parcels in three bilateral networks, though most participants demonstrated greater sensitivity to parcels in the left hemisphere (see Figure 5; Table 3).

For all eight participants, significant differences were found at the *p* < 0.05 significance level between HbO parcel values calculated using the subject-specific and generic optode locations. For frontal recordings, >84% of parcels at the individual level demonstrated *p*-values (FDR-corrected) below the significance level. For motor and visual recordings, the percentage range for parcels with *p*-values below the significance level was >71 and >86%, respectively. At the group level, significant differences in HbO parcel values at the *p* < 0.05 significance level were seen in a higher percentage of parcels than at the individual level. For group-averaged frontal measurements, 95.7% of parcels had *p*-values below the significance level. For group-averaged motor and visual measurements, this percentage was 84.4 and 96.4%, respectively.

To assess whether there was a relationship between parcel size and parcel sensitivity, we plotted the average parcel sensitivity percentage against the total number of nodes comprising each parcel (see Supplementary Figure 2). For each of the three recording types, no clear relationship between parcel size and sensitivity could be determined.

### Generic optode locations overestimate cortical sensitivity

In general, using the generic optode locations led to an overestimation of the NIRS array sensitivity to the cortical parcels. In Tables 1–3, the difference in sensitivity percentage is shown, calculated as the difference between the subject-specific and generic models. There are more negative values for the percent difference in sensitivity, indicating that parcel sensitivity for the generic model was systemically higher than that of the subject-specific model. For both the motor and the visual recordings, ~58% of sensitivity percentage differences are due to an overestimation of cortical sensitivity using the generic optode locations. In addition, for the motor recordings, it was found that the number of parcels that ≥75% of subjects were sensitive to using the subject-specific array was 23, while with the generic array, the number of sensitive parcels was overestimated to be 31. In particular, the generic motor array demonstrated sensitivity to parcels in the superior parietal lobule (left hemisphere: *n* = 1 parcel), inferior parietal lobule (left hemisphere: *n* = 8 parcels), intraparietal sulcus (left hemisphere: *n* = 4 parcels), post central gyrus (right hemisphere: *n* = 10 parcels), and precentral gyrus (right hemisphere: *n* = 2 parcels) across at least 75% of participants, all of which the subject-specific arrays did not demonstrate any sensitivity to. Furthermore, large variations in sensitivity (>50% difference in parcel sensitivity) were seen in multiple participants, particularly for motor parcels (bolded entries in Tables 1–3).

**Table 1:** Differences in motor parcel percent sensitivity observed when using the subject-specific optode locations compared to the generic optode locations.

**Hemisphere**	**Network (parcel)**	**% difference in sensitivity (subject-specific—generic model)**							
		**P1**	**P2**	**P3**	**P4**	**P5**	**P8**	**P9**	**P10**
Left	Somatomotor A	−4.49	−15.58	−8.61	−17.42	−10.86	–**23.19**	−21.68	−7.35
Right		–**30.26**	−19.49	−29.63	−13.16	−2.79	−11.97	−13.30	−5.81
Left	Dorsal Attention B (post central)	11.42	7.72	23.70	−35.02	−1.32	–**59.50**	−49.50	0.00
Left	Salience/ventral attention A (parietal operculum)	20.63	12.47	−0.08	−16.00	3.89	–**26.12**	−12.20	0.00
Left	Salience/ventral attention B (inferior parietal lobule)	40.00	19.36	0.00	−8.33	11.67	–**58.33**	−56.67	0.00
Right		9.46	0.00	3.99	−17.86	**27.92**	0.00	−27.44	0.00
Left	Control A (intraparietal sulcus)	12.07	−4.84	12.18	−39.23	−26.18	–**40.00**	–**40.00**	0.00
Right		–**32.36**	0.00	3.99	−17.86	27.92	0.00	−27.44	0.00
Left	Control B (inferior parietal lobule)	−4.33	−16.95	−20.51	−65.70	−17.23	−66.10	–**67.78**	0.00
Right		−15.02	0.00	−12.70	−54.13	−32.28	0.00	–**54.51**	0.00
Left	Control B (lateral PFC)	4.65	−1.96	10.85	58.81	−3.14	**65.72**	48.84	7.82

Parcels listed in the table are those that at least half (4/8) of the participants demonstrated sensitivity to. The maximum sensitivity difference for each parcel is shown in bold.

**Table 2:** Differences in prefrontal parcel percent sensitivity observed when using the subject-specific optode locations compared to the generic optode locations.

	**Network (Parcel)**	**% difference in sensitivity (subject-specific—generic model)**							
		**P1**	**P2**	**P3**	**P4**	**P5**	**P8**	**P9**	**P10**
Left	Limbic B (orbitofrontal cortex)	−2.97	−9.13	0.68	−10.25	−11.04	−15.43	–**16.97**	−4.68
Right		−4.01	−14.36	–**17.04**	−12.84	−14.93	−15.47	−14.16	−2.79
Left	Control B (dorsal PFC)	−9.61	0.00	−12.47	21.86	19.78	14.96	17.68	**26.82**
Left	Control B (lateral PFC)	0.39	0.00	**22.91**	12.08	17.56	11.67	2.35	11.76
Left	Control B (lateral ventral PFC)	−2.24	23.36	14.26	−10.88	−12.64	**78.34**	−37.36	−8.95
Right		−18.05	24.94	5.92	−0.41	−20.62	−23.02	−24.92	–**29.36**
Left	Default A (dorsal PFC)	−6.80	0.40	−9.92	−21.69	−16.50	12.50	12.50	–**34.10**
Right	Salience/ventral attention B (lateral PFC)	−5.41	−32.61	**41.28**	4.92	11.04	0.00	0.00	18.19
Right	Default B (ventral PFC)	−9.06	−12.42	**26.24**	−15.36	−21.81	−10.61	−17.13	−14.16

Parcels listed in the table are those that at least half (4/8) of the participants demonstrated sensitivity to. The maximum sensitivity difference for each parcel is shown in bold.

**Table 3:** Differences in visual parcel percent sensitivity observed when using the subject-specific optode locations compared to the generic optode locations.

**Hemisphere**	**Network (parcel)**	**% difference in sensitivity (subject-specific—generic model)**							
		**P1**	**P2**	**P3**	**P4**	**P5**	**P8**	**P9**	**P10**
Left	Visual central (striate cortex)	0.44	−0.85	−10.33	−20.43	0.00	3.71	−27.26	–**72.34**
Right		−19.71	−8.58	−5.89	−2.90	0.00	–**26.45**	−15.12	−21.76
Left	Dorsal attention A (parietal cortex)	−11.36	−8.99	15.38	8.04	16.49	6.36	**28.84**	0.00
Left	Default C (inferior parietal lobule)	−32.52	0.00	28.16	15.20	0.00	12.75	**35.56**	0.00

Parcels listed in the table are those that at least half (4/8) of the participants demonstrated sensitivity to. The maximum sensitivity difference for each parcel is shown in bold.

## Discussion

To our knowledge, this study is the first to investigate regional differences in optode localization error and anatomical sensitivity using HD-DOT data. Using an atlas-guided parcellation method for performing group-level analysis, as shown in Figure 1, we have demonstrated that subject-specific optode localization reveals large cortical sensitivity differences between subjects (Tables 1–3) which must be taken into account when performing region-of-interest analyses at the group level, to ensure that equivalent cortical regions have been reliably sampled across subjects.

We have found that the brain region being imaged (frontal, motor, or visual) greatly impacts the degree of error between subject-specific optode locations and generic optode locations. These brain regions were selected to replicate the most used regions in fNIRS studies. Additionally, we have shown that for the same HD-DOT array, cortical sensitivity varies substantially between participants and is largely overestimated when the same generic optode locations are assumed for all participants. This is particularly important for HD-DOT studies that perform group-level analysis, to ensure comparability across subjects.

### Variation in optode placement across the head

As demonstrated in Figure 2, while there were large variations in the optode localization error between the generic and subject-specific maps across the head, the median errors for both the motor and frontal HD-DOT array placements were non-trivial when compared to the smallest source-detector distance of the LUMO system (~10 mm between adjacent light sources and photodetectors on the same tile). Only the median error for the visual array was smaller than the shortest channel length, though the maximum error for this array (9.72 mm) was very close to 10 mm. The lower median error for the visual array may be partly attributed to the smaller area covered.

Furthermore, for the motor array placement, the median error was found to be just under 30 mm, which is the standard length of a NIRS channel used for recording from the cortex. This highlights the importance of subject-specific optode localization and standard placement across participants to ensure that accurate group-level comparisons can be made. Variabilities in channel coverage at this scale may lead to the association of brain activity originating from adjacent non-motor regions to the motor cortex, or vice versa. In particular, we found that our motor array demonstrated high sensitivity across participants to post central and precentral ventral parcels in the dorsal attention network, which are adjacent to the somatomotor networks, and can result in the misattribution of activity in these areas to the motor cortex if care is not taken to perform subject-specific optode localization. These results are in line with previous findings in infants that variabilities in array positioning are the primary factor behind different anatomical inferences at both the individual-level and the group-level for small group sizes (Collins-Jones et al., 2021).

Variations in optode placement are important for both fiber-based and fiberless systems, however the impact is likely more severe in fiberless systems where the geometry of the sources and detectors is pre-defined (e.g., Chitnis et al., 2016; Zhao et al., 2020). For modular, fiberless systems in particular, such as the one used in this study, the rigidity of the sensor modules and the dock layout of the cap may limit variations in optode placement and make it difficult to align optodes consistently across subjects. This device design may partially contribute to the uncertainties in generic optode positions across subjects. Fiber-based HD-DOT systems could mitigate this issue, though the optodes are still typically integrated into a cap which may again limit consistent subject-specific optode alignment, albeit to a lesser degree than modular systems (e.g., Eggebrecht et al., 2014; Fishell et al., 2020). However, even for traditional fiber-based fNIRS studies, where optodes can be individually placed on the scalp surface, typically according to the international 10–20 positioning system, the spatial identity of a given channel across participants should be verified to ensure that the same cortical volume is being recorded from. For example, in fNIRS-based brain-computer interfaces (BCIs), where recording over the primary motor cortex is most common (Naseer and Hong, 2015), accounting for inter-subject variability by performing optode localization may lead to more robust and accurate classification. This is particularly true for generic fNIRS BCIs, where classification models are trained to identify different hemodynamic signals across subjects (as opposed to being tailored to individual subjects), given that one of the largest challenges for subject-independent BCIs is the presence of inter-subject variability (Abdalmalak et al., 2020). As has been demonstrated in EEG-based BCI literature, accounting for variation in individual electrode positioning and head anatomy can lead to classification improvements using generic models (Wronkiewicz et al., 2015). Image reconstruction results for HbO, shown in Supplementary Figure 3, further demonstrate the difference in spatial activity maps for the motor cortex when using the generic and subject-specific optode locations at the group level.

The lateral and frontal views in Figure 2A demonstrate that the highest errors in optode localization occur along the sides of the head and above the brow ridge. This is potentially due to larger variability between participant head shapes in these areas, possibly as a function of greater curvature in these regions. For each participant, the LUMO cap was tightened using an adjustable chin strap to ensure adequate tension throughout the cap and good optical coupling across all optodes. As we did not observe lower optical coupling in the regions that demonstrated higher errors in optode localization, we believe that our results are a function of the variability in head curvature as opposed to the stress distribution of the cap design. This is also supported by previous work that demonstrated slightly larger prediction errors around the brow bone and above the ears when attempting to generate subject-specific head models using three-dimensional adult head scans and anatomical landmarks (Park et al., 2021). It is also important to note that in this study, the same cap (56–58 cm head circumference) was used for all participants. In practice, different cap sizes may be used for different individuals, depending on the size of the individual's head, to ensure optimal fit during HD-DOT scanning.

### Between-participant differences in cortical sensitivity

In the parcellation analyses presented, we aimed to assess the degree to which cortical sensitivity between participants varies as a function of array positioning, as well as assessing the validity of assuming generic optode locations when performing anatomical parcellation. For all three HD-DOT arrays evaluated, multiple parcels, that at least half of the participants showed sensitivity to, had large differences in sensitivity when using subject-specific and generic optode locations. We found that these differences were largest for the motor array, followed by the frontal array, both of which are consistent with our findings for the largest optode localization errors. In particular, while we found that using the generic optode locations leads to an overestimation of cortical sensitivity across the brain regions measured, they also demonstrated more uniform cortical sensitivity between the left and right hemispheres. By contrast, the use of subject-specific optode locations demonstrated resting state lateralization, particularly for the frontal array. Our results are consistent with findings of lateralization in resting state networks from fMRI literature (Agcaoglu et al., 2015). Additionally, the most strongly lateralized networks identified with resting state fMRI were found to be the visual, default, and attentional networks (Liu et al., 2009). This may explain why we also saw larger differences in parcel sensitivity between the subject-specific and generic optode locations within these networks. In particular, we found a lower Jaccard index for the visual recordings, which is likely due to strong lateralization in the resting state visual network, something which we observed with the subject-specific, but not generic, visual optode array. We also found that for both the frontal and motor arrays, the mean difference in sensitivity percentage was higher for parcels in the control networks (see Tables 1, 2). This is again in line with our findings for optode localization error, as the majority of sensitive parcels in these networks lie within the regions where the greatest optode localization error was found (see Figures 3, 4), further outlining the need to accurately relate scalp locations with the cortical area underneath.

We initially investigated different sensitivity thresholds to assess whether greater agreement between the generic and subject-specific optode array sensitivities could be achieved simply by changing this value. With a lower sensitivity threshold (2.5%), we observed that array sensitivity to any given parcel increased, along with the total number of parcels we were sensitive to, demonstrating slightly greater agreement between the two methods. However, we still found that the generic optode array systemically overestimated sensitivity to a majority of parcels compared to the subject-specific optode array. As a result, we chose to present our results using the 5% threshold, as defined in the literature (Uchitel et al., 2022), as a conservative measure of array sensitivity.

In this study, we have measured resting state data across three brain regions, primarily to record intrinsic, rather than task-related, activity in the brain. Doing so allowed us to match parcels to resting state networks defined from fMRI, in order to assess not just the anatomical regions we are sensitive to, but also the functional areas. One common misunderstanding may arise from the use of anatomical parcels registered to resting state networks: While the resting state networks refer to the cortical regions that exhibit synchronous activity, as measured in the resting state, the parcels simply refer to anatomical areas, independent of resting or task-related activity. Measuring resting state data reduces the risk of task-based confounding factors leading to the under- or over-estimation of cortical sensitivity (Fox and Greicius, 2010). Additionally, when measuring resting state data, spontaneous changes in hemodynamic response are measured over the entire cortex, without having to design tasks that localize activity to a particular region of interest (Lee et al., 2013; Huang, 2019).

It has been shown that, in the human brain, there are approximately 400 distinct cortical areas (Van Essen et al., 2012). Thus, while a lower resolution parcellation such as the 400-parcel Schaefer atlas may be sufficient for certain applications and may mitigate some of the error introduced by optode array variability, owing to the larger parcel volumes in lower resolution parcellation atlases, we instead chose to use the 1,000-parcel Schaefer atlas to avoid oversimplifying the high-density spatial data that we have collected. Additionally, we found that parcel size was not related to parcel sensitivity (see Supplementary Figure 2), meaning that errors in parcel sensitivity are likely due to differences in array positioning rather than the size of the individual parcels themselves.

## Limitations and future work

This study demonstrates the importance of collecting subject-specific anatomical and array placement information for fNIRS studies, particularly HD-DOT studies, in order to accurately draw conclusions about the anatomical regions where functional responses are seen. The identification of these regions also allows for direct comparison of fNIRS findings to those from more prevalent functional imaging techniques such as fMRI.

However, one limitation of this work is that a standard head template was used, under the assumption that cortical structure for healthy participants could be adequately represented by the MNI152 template. The use of subject-specific MRI data to develop individual head models and perform image reconstruction has been demonstrated to reduce the localization error in DOT by a factor of two (Cooper et al., 2012). While atlas-guided head modeling has been shown to demonstrate slightly higher errors compared to subject-specific MRIs (Ferradal et al., 2014), Custo et al. (2009) found that with a high-resolution atlas, spatial precision at the gyral/sulcal-level was achievable in DOT image reconstruction. Nevertheless, future studies should consider evaluating the error reduction achievable when using subject-specific MRIs in conjunction with subject-specific optode localization, as well as the performance of non-linear registration methods, which have been established as more accurate alignment methods than simple affine transformations (Klein et al., 2009). While the findings we have presented can be extended in multiple ways, we believe that we have verified the need to collect subject-specific optode locations for wearable fNIRS experiments, particularly HD-DOT studies, in order to perform accurate group-level analysis to then be able to relate brain structure and function.

## Data availability statement

The raw data supporting the conclusions of this article will be made available by the authors, without undue reservation.

## Ethics statement

The studies involving humans were approved by Cambridge School of Technology Research Ethics Committee. The studies were conducted in accordance with the local legislation and institutional requirements. The participants provided their written informed consent to participate in this study.

## Author contributions

SS: Conceptualization, Data curation, Formal analysis, Investigation, Methodology, Software, Writing—original draft, Writing—review & editing. DA: Conceptualization, Data curation, Investigation, Writing—review & editing. EB: Data curation, Writing—review & editing. LC-J: Formal analysis, Methodology, Writing—review & editing. FM: Supervision, Writing—review & editing. GB: Conceptualization, Supervision, Writing—review & editing.
