# Supplementary material for: Subject-specific information enhances spatial accuracy of high-density diffuse optical tomography
Source: Front Neuroergon. 2024 Feb 19;5:1283290. doi: 10.3389/fnrgo.2024.1283290 (PMC10910052; doi:10.3389/fnrgo.2024.1283290)
Supplement: Supplementary file 1 [file Data_Sheet_1.pdf]

Supplementary Material

Subject-Specific Information Enhances Spatial Accuracy of High-Density Diffuse Optical Tomography

Sruthi Srinivasan*, Deepshikha Acharya, Emilia Butters, Liam Collins-Jones, Flavia Mancini, Gemma Bale

*** Correspondence:** Corresponding Author: ss2814@cam.ac.uk

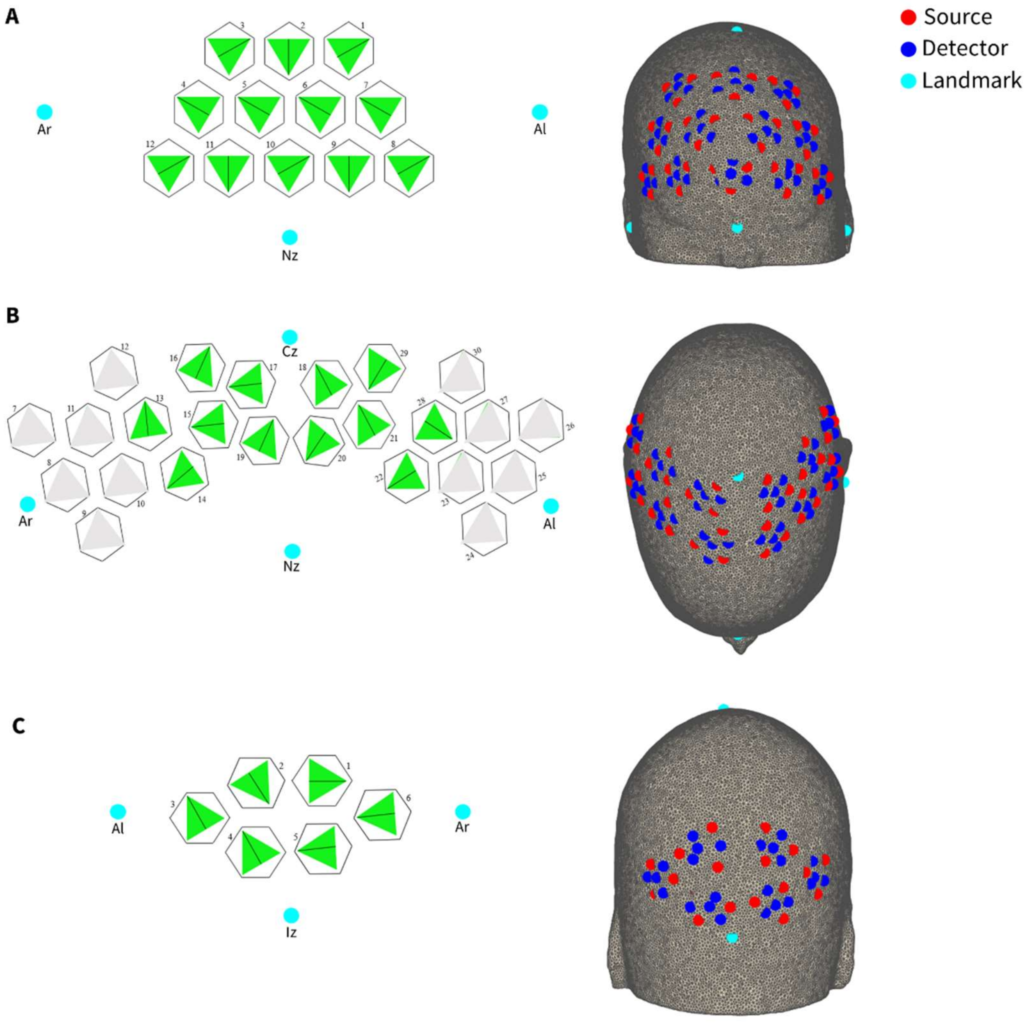

Supplementary Figure 1. LUMO tile layouts (left) and source/detector positions registered to a standard head model derived from the MNI152 template (right) for **(A)** frontal, **(B)** motor, and **(C)** visual resting state recordings. Grey triangles in tile layouts represent empty docks on the LUMO cap.

Supplementary Table 1. Participant head sizes, defined by the distance between the inion and nasion and the distance between pre-auricular points. These geodesic distances were calculated from the head models of each participant (derived by an affine transformation of the MNI152 atlas, performed using subject-specific cranial landmarks).

Participant	Inion to Nasion Distance (cm)	Pre-Auricular Right to Pre-Auricular Left Distance (cm)
1	35.38	35.26
2	36.57	37.35
3	35.53	37.85
4	38.66	39.65
5	38.46	39.76
6	35.13	33.77
7	37.22	36.92
8	36.78	38.69
9	41.12	41.11
10	38.62	37.40

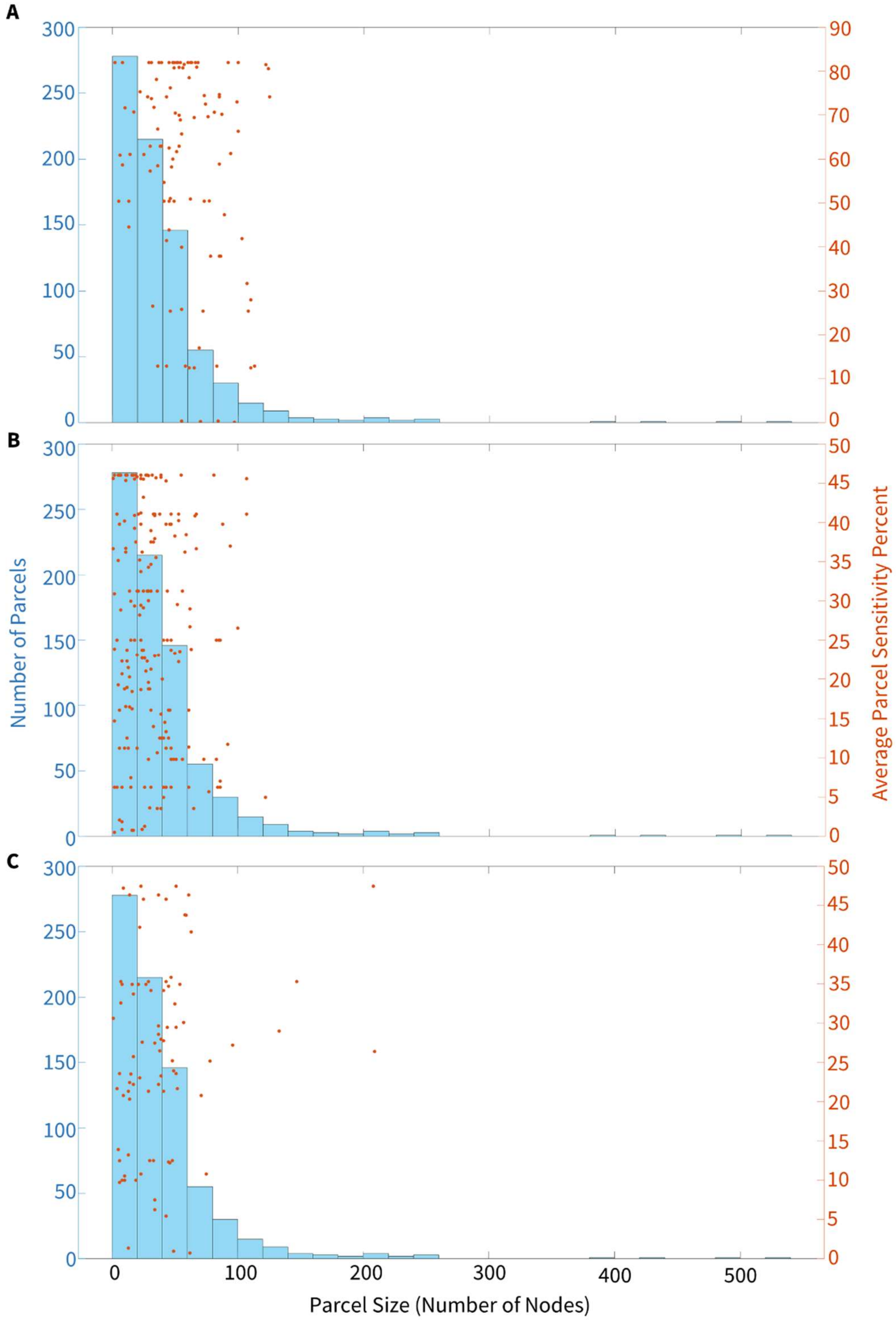

Supplementary Figure 2: Relationship between parcel size (defined by the number of nodes a parcel is comprised of) and the mean parcel sensitivity across participants, using subject-specific optode locations. The underlying histogram shows the overall distribution (left axis) of parcel sizes in the Schaefer 1000-parcel atlas, while red points represent the mean percentage sensitivities (right axis) for the specific parcels reached by the **(A)** frontal, **(B)** motor, and **(C)** visual HD-DOT arrays.

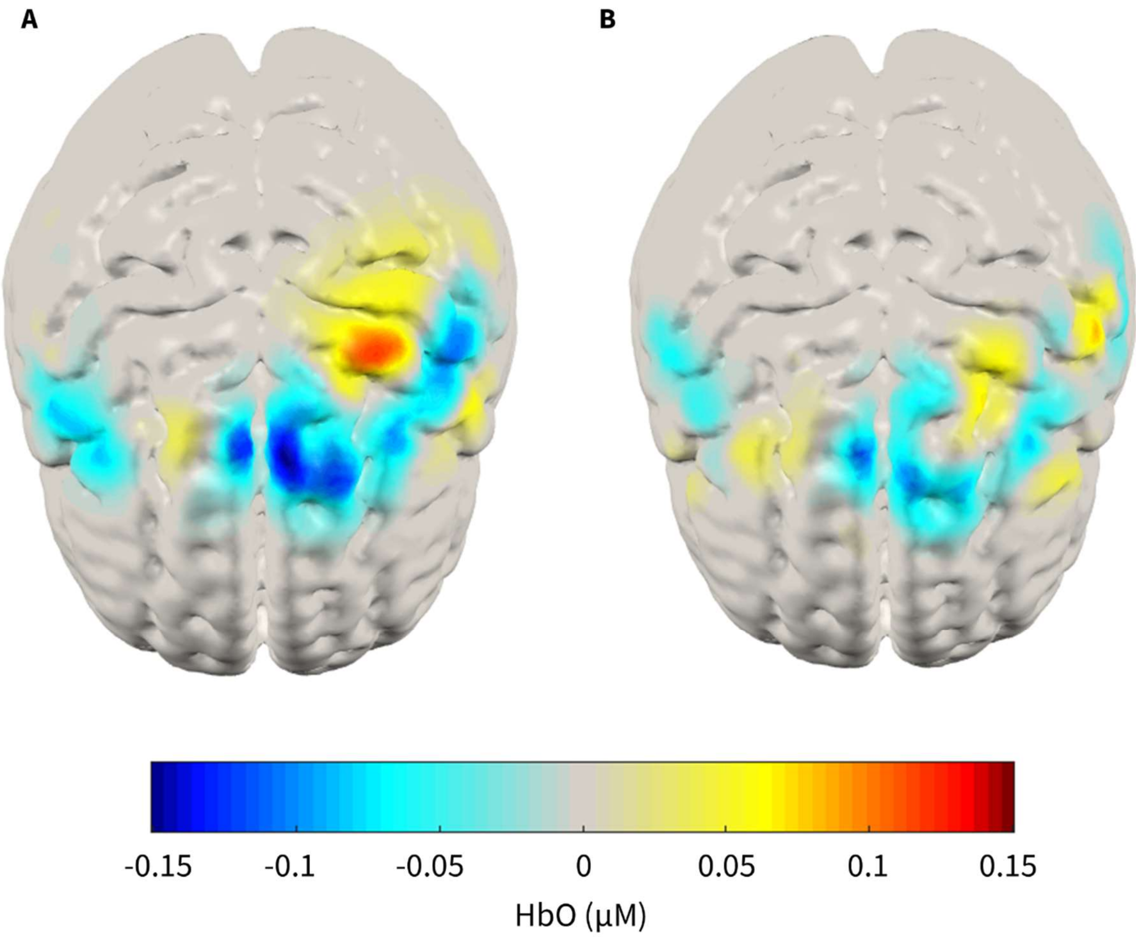

Supplementary Figure 3: Differences in group-averaged, HbO image reconstruction results for the motor cortex (superior view), shown for $n = 8$ participants. Changes in HbO concentrations, averaged over the duration of the recording period (60s), are derived from image reconstruction using **(A)** generic and **(B)** subject-specific optode locations.
